# Dr. Sanden

**Published:** 1840-10

**Authors:** 


### OBITUARY.

### THOMAS SANDEN, M.D.

[THE two following articles, written by intimate friends of the deceased, give so faithful a picture of the venerable and beloved man therein commemorated, that we gladly transfer them to our pages, without alteration or comment. The first is extracted from No. lxxxi. of "The Christian Reformer;" the last from "The Hampshire Telegraph," of the 13th July, 1840.]

I.

Died, July 11, at Chichester, Thomas Sanden, M.D. Dr. Sanden was born in that city on the 24th of February, 1752, and, excepting the period of his school and college education, spent the whole of his life there. His father held the chief office in the Custom-house at Chichester, and was an alderman of the city. Dr. Sanden received his education at the academy of the Rev. S. Merivale, at Exeter, from whence he went to the university of Edinburgh, where he took the degree of M.D. in 1774; his thesis, distinguished alike for its latinity and learning, being *De Atmosphæræ natura et effectibus quibusdam* He returned to Chichester in the year 1775, and practised there as a physician for more than fifty years. He was for great part of that time the only physician in Chichester; his practice was very considerable in the city and its neighbourhood; and he was not unfrequently called upon to give his medical assistance at a considerable distance from his residence. The generosity of his character, however, prevented his receiving more than a very moderate remuneration from his extensive practice. His gratuitous services were often given to a very considerable extent. About the year 1784, in conjunction with a benevolent clergyman of the established church, the late Rev. W. Walker, he projected and succeeded in establishing a Dispensary at Chichester. At a much later period, he supported a plan brought forward by his friend and brother physician, Dr. Forbes, of erecting an Infirmary there; and he had the happiness to witness the complete success of this undertaking. Dr. Sanden's character as a physician was duly appreciated by those who were most competent to form a correct judgment; and the highest testimony to his medical skill and science was repeatedly given by the late celebrated Dr. Matthew Baillie, with whom he had occasionally very agreeable and friendly intercourse. He was one of the founders of the Literary Society at Chichester, and for many years its president.

In the year 1782, he was married to Miss Grace Poole, sister of the late Rev. Sir Henry Poole, of the Hook, near Lewes; a lady possessing every quality of the head and heart which could ensure matrimonial felicity. They lived together fifty years in the closest bonds of esteem and affection. She died in the year 1832. They had no children.

The mind of Dr. Sanden was of a decidedly superior order. His apprehension was clear and distinct, his judgment sound, his reasoning power strong, his taste refined and elegant. His knowledge and skill in his profession have been already adverted to. He was well acquainted with several branches of science, and his mind ranged over almost the whole extent of literature. To classical literature he had given much attention; poetry and the *belles lettres* greatly interested him; but he more particularly devoted himself to theological, metaphysical, and ethical studies. He had embraced early in life those religious opinions which are commonly called Unitarian, and he continued strongly attached to them as long as his faculties remained. In philosophy, he was an admirer and follower of Locke, Hartley, Priestley, and Paley. He regarded Locke with the highest respect, as the father of rational metaphysics; and he fully adopted the metaphysical theory of Hartley. He agreed in nearly all the philosophical and theological opinions of Priestley, for whose character he entertained great esteem; but for many years before his death he had ceased to think highly of the judgment of that great and excellent man. He estimated highly the writings of Paley. In politics, he was a decided and moderate Whig; sincerely attached to the British constitution, but anxious to forward every real reform, and to improve the institutions of his country. It is certain that he thought highly of the Philosopher of Malmesbury, the embracing of whose opinions seemed to many of his friends inconsistent with his general principles. He published a vindication of his sentiments respecting the doctrines of Hobbes, in a series of letters, with the signature of Hylas, in the Monthly Repository.

He led an extremely retired and studious life, and occasionally, but by no means frequently nor to any great extent, gave his thoughts to the public. The following is a list of his principal publications:

Miscellaneous Observations on some points of the Controversy between the Materialists and their Opponents. 1780.

Short Strictures on the Method of Treatment, recommended by Dr. Dawson in Acute Rheumatism. 1782.

A Letter to the Bishop of St. David's, occasioned by his Sermon on the Principle of Vitality in Man. 1789.

Select Parts of the Introduction to Dr. Gregory's Philosophical and Literary Essays, methodically arranged and illustrated with Remarks, by an Annotator.

Three Discourses on the Use of Books, delivered to the Literary Society at Chichester in the years 1800, 1801, and 1802.

Singular Termination of a Case of Enteritis, (Annals of Med. VI.) 1801.

Two Letters to Dr. Goddard. 1811, 1815.

A Tribute to the Memory of John Bayly, M.D. 1816.

A short Biographical Memoir of Thomas Potter Powell, M.D., prefixed to a Sermon on his Death. 1816.

Unitarianism, Old and New. 1817.

Letters signed Hylas (already mentioned.)

His style as a writer was very perspicuous, pure and elegant; and he expressed himself in conversation with singular neatness and accuracy of language, though not without considerable hesitation. His manners were those of a perfect gentleman of the old school. He was what would be considered in the present day too ceremonious; and there was an appearance of reserve and coldness in his communications with his nearest friends. This, however, was mere outside; his friendships were in reality peculiarly warm. No man was more averse to giving pain. Humility was a leading trait in his character; and perhaps his usefulness was in some measure restricted by a want of that confidence in himself to which his abilities and knowledge entitled him. Nevertheless, he was capable (when his natural diffidence could be overcome) of acting with great decision and firmness. His benevolence extended to all, and his bounty was largely distributed among those who needed his assistance. A deep-seated and heart-felt piety was the leading feature of his character. His views of religion were most cheering; he saw God in everything, and believed that all events are regulated by Divine Providence.

For the last five years of his life he had been confined to his bed; his faculties had failed by degrees; and for a considerable time he had ceased to know his friends, and had become quite unconscious of everything about him. The powers of life at length gave way, and he tranquilly yielded up his breath, in the eighty-ninth year of his age.

II.

Died, on Saturday, the 11th of July, at his residence in the South Pallant, Chichester, in the eighty-ninth year of his age, Thomas Sanden, M.D., a man of rare intellectual endowments, and of yet rarer virtues. He practised as a physician in his native city for upwards of fifty years, and with a degree of skill, liberality, and kindness which acquired for him the love and veneration of the whole community. Dr. Sanden's time of birth and education placed him in the class of the older English physicians, who possessed a knowledge of classical literature and of the medicine of the ancients, rarely acquired by the practitioners of the present day; while his active and liberal spirit, totally free from the prejudices of age or sect, kept his acquirements constantly on a level with the modern improvements in medical science. The result was that Dr. Sanden was one of the most scientific and successful physicians of his time. His learning was great and varied, embracing the whole field of science and literature. His favorite studies, extra-professional, were metaphysics and theology, in both of which he was profoundly versed. His published writings, which were for the most part anonymous, prove him to have possessed a most refined literary taste, and an acute and logical mind. In the moral part of his nature, in his conduct as a member of society, and in all the relations of life, Dr. Sanden was, probably, as nearly faultless as falls to the lot of frail mortals. In affection for his fellow-men of all races and ranks, in the desire to do good and eschew evil, in charity and liberality in the interpretation of the words, actions, and motives of all, in love of God and in profound veneration of His goodness and superintending mercy, Dr. Sanden was excelled by no man; and rarely has there descended to the grave a purer spirit, or one who, through a long and active life, more constantly, devotedly, and sincerely followed the Christian precepts of doing as we would be done unto, of loving all men as brothers, of cherishing in the inmost heart a charity that beareth all things, and forgiveth all things, and hopeth all things. There was a defect in Dr. Sanden's intellectual constitution which, in some degree, interfered with his usefulness as a member of society. His extreme modesty and self-depreciation led to a want of boldness in action which often rendered ineffective what his benevolence planned and his judgment approved. The same qualities confined him, through his whole career, to the quieter shades of life—to the *tranquilla silentia vitæ*, as he himself expressed it; and it was, in consequence, only his more intimate friends who knew the full extent of his mental endowments and acquirements, and his almost matchless virtues. By such friends he was loved and venerated with a warmth which cannot well be measured and which will endure with memory.

